# Science for the Next Century: Deep Phenotyping

**DOI:** 10.1177/00220345211001850

**Published:** 2021-03-20

**Authors:** J.T. Wright, M.C. Herzberg

**Affiliations:** 1 Adams School of Dentistry, University of North Carolina at Chapel Hill, Chapel Hill, NC, USA; 2 Department of Diagnostic and Biological Sciences, School of Dentistry, University of Minnesota, Minneapolis, MN, USA

**Keywords:** phenotype, genotype, craniofacial, microbiome, caries, periodontal

## Abstract

Our ability to unravel the mysteries of human health and disease have changed dramatically over the past 2 decades. Decoding health and disease has been facilitated by the recent availability of high-throughput genomics and multi-omics analyses and the companion tools of advanced informatics and computational science. Understanding of the human genome and its influence on phenotype continues to advance through genotyping large populations and using “light phenotyping” approaches in combination with smaller subsets of the population being evaluated using “deep phenotyping” approaches. Using our capability to integrate and jointly analyze genomic data with other multi-omic data, the knowledge of genotype-phenotype relationships and associated genetic pathways and functions is being advanced. Understanding genotype-phenotype relationships that discriminate human health from disease is speculated to facilitate predictive, precision health care and change modes of health care delivery. The American Association for Dental Research Fall Focused Symposium assembled experts to discuss how studies of genotype-phenotype relationships are illuminating the pathophysiology of craniofacial diseases and developmental biology. Although the breadth of the topic did not allow all areas of dental, oral, and craniofacial research to be addressed (e.g., cancer), the importance and power of integrating genomic, phenomic, and other -omic data are illustrated using a variety of examples. The 8 Fall Focused talks presented different methodological approaches for ascertaining study populations and evaluating population variance and phenotyping approaches. These advances are reviewed in this summary.

## Introduction

Amazing advances have been made in our ability to acquire and evaluate human genomics information since the publication of the first draft of the human genome (Venter et al. 2001; Green et al. 2020). Health and disease are increasingly understood to be determined by many factors, including genetics, transcriptomics, proteomics, metabolomics, microbiomics, and lifestyle and environmental determinants and modifiers. Understanding the human genetic code, underlying molecular mechanisms, and environmental determinants is foundational for defining and delineating phenotypic variance associated with genotype-phenotype relationships in both health and disease (Delude 2015). This knowledge is critical for advancing predictive and precision-directed health care and health outcomes (Robinson 2012; Delude 2015). Extending our understanding of phenotypes to more granular levels (e.g., deep phenotyping) and without the constraint of narrowly defined disease phenotypes is improving our understanding of health and pathophysiology through life (Tracy 2008). Challenges remain, however, to the characterization of complex clinical phenotypes. New technologies and approaches are advancing our ability to define, measure, and quantify phenotypes, thereby aiding our ability to develop new knowledge about the dental, oral, and craniofacial complex.

## Deep Phenotyping

**Figure 1.**
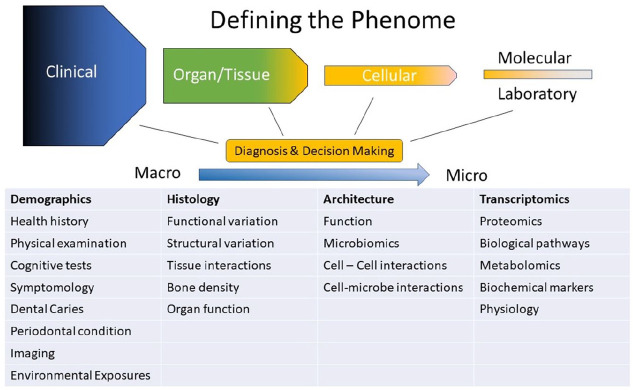
Defining the oral, dental, and craniofacial phenome involves the observation and assessment of parameters that range from macro-phenotyping to micro-phenotyping. Light phenotyping may involve a small subset of traits, whereas deep phenotyping is more detailed and granular phenotyping.

Deep phenotyping is the precise and comprehensive analysis of observable traits that are a consequence or outcome of genetics, epigenetics, lifestyle, and environmental influences. Deep phenotyping requires the acquisition of information characterizing the span from clinical to molecular parameters (Fig. 1). The use of a standardized, controlled vocabulary allows phenotypic information to be described and communicated in an unambiguous fashion. This vocabulary facilitates consistent descriptions of features observed in medical settings, publications, and databases. A phenotypic vocabulary and curated disease-phenotype annotation is being compiled in the Human Phenotype Ontology (Kohler et al. 2017; Mishra et al. 2019).

To advance public health, precision health care must move toward an operational reality. This reality is fostered by improved genotype and phenotype methodologies and cost reductions in genomic analysis. As compared with the more traditional single-trait analyses, deep phenotyping with simultaneous genome-wide association analyses serves as a discovery tool to reveal previously unsuspected relationships between phenotypic traits and with specific molecular mechanisms (Bycroft et al. 2018). Elucidation of these relationships will certainly improve diagnostics, understanding of disease and pathogenesis, and ultimately therapy.

To present the state of the art, the American Association for Dental Research presented “Science for the Next Century: Deep Phenotyping” as a Fall Focused Virtual Webinar. During 4 sessions in fall 2020, the program consisted of expert talks on deep phenotyping with examples ranging from dental caries, periodontal disease, and the microbiome to dental and craniofacial anomalies. These presentations and the importance of these scientific advances in dental, oral, and craniofacial research are now highlighted.

## Genotype-Phenotype Study Design

In the opening session, Dr. Leslie Biesecker discussed the traditional health care approach, which focuses on a sick patient or individual with a phenotype. Patients are evaluated with the goal of making a diagnosis and providing appropriate treatment. A contemporary alternative is based on the availability of low-cost, high-throughput genomic analysis. Genomic analyses facilitate a predictive medicine approach by evaluating genotypes of individuals. Precise genotypic characterization is thought to better instruct early preventive or therapeutic interventions (Biesecker et al. 2009). These technological advances and changing approaches to diagnosis and patient evaluation are creating an evolution in the health care model and the approach to disease management.

**Figure 2.**
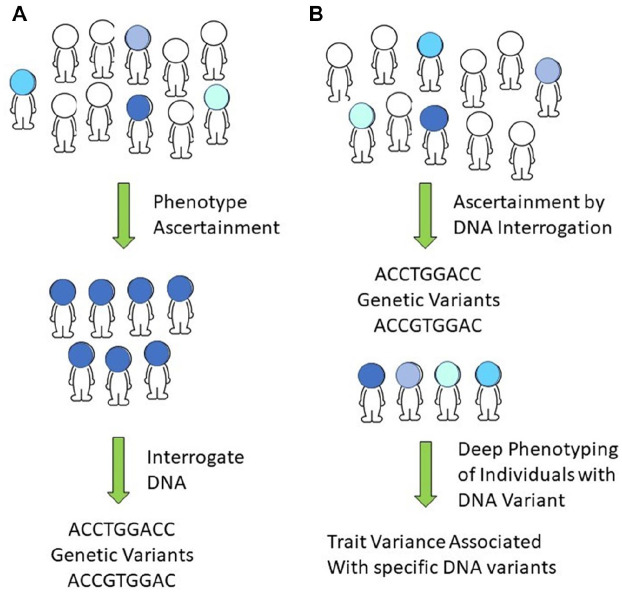
Diagnosis of disease has traditionally involved identifying a specific disease phenotype and then finding associated DNA variation (**A**). This approach identifies individuals with a specific phenotype and can exclude individuals who deviate from the specific ascertainment phenotype. Alternatively, we can identify genetic variants in the general population, allowing inclusion of all individuals with the genetic variant of interest regardless of the resulting phenotype (**B**). This approach has the benefit of being able to identify phenotypic variability associated with specific DNA alterations that can be missed using a phenotype population approach.

Dr. Biesecker and Dr. Kimon Divaris addressed the value of evaluating the phenotype and genotype of populations rather than individuals. Evaluating individuals recruited for study based on phenotype causes an inherent bias based on the a priori assumption of the phenotype of interest if enrollment is based on phenotype screening. Alternatively, populations can be assessed based on a genotype of interest. Individuals can be ascertained with specific genetic variants, which allows focused deep phenotyping and characterization of specific molecular changes that help define the clinical variance associated with a specific genotype (Fig. 2; Patton et al. 2016). After genotyping, study participants can be recalled for careful phenotypic evaluation, such as in the ClinSeq study (Sloan et al. 2011). Unfortunately, many large-scale population genotyping initiatives did not plan for further phenotype assessment, limiting the utility for analysis of a specific phenotype. Many population-based studies included minimal self-reported dental and oral phenotyping of questionable accuracy. These studies can provide limited but potentially useful information about specific dental and oral genotype-phenotype relationships in an efficient manner (Divaris 2019). Importantly, to advance our understanding of the genetic basis of diseases and their biological intermediates, we must identify and assess the disease process (e.g., dysbiotic microbial communities, dysregulated inflammatory response) and not solely clinically manifest disease outcomes (e.g., caries lesions, gingival attachment loss).

## Dental Caries

Refining the caries phenotype is key to unraveling the complex biological relationships that influence the process of biofilm-mediated dental hard-tissue demineralization. Dental caries and periodontal disease are person-level disease processes that are heterogenous between surfaces, individuals, and populations and require an understanding of the tooth-biofilm interface, behaviors, environment, and host genetic variation. Understanding the biological process is critical to appreciating and eventually leveraging the diverse clinical phenotypes. Commonly used clinical measures of dental caries, such as the numbers of decayed, missing, and filled surfaces, describe the outcome of the process but do not assess the process itself. The oral microbiome is extremely diverse with about 2000 bacterial, archaeal, viral, and fungal species; dental caries can result when the plaque biofilm community becomes dysbiotic (Sampaio-Maia et al. 2016). Initial studies by Dr. Divaris used latent class analysis and identified differing microbial profiles associated with distinct clinical patterns of early childhood caries or disease subtypes. The presence of caries subtypes has been previously suggested for adults (Shaffer et al. 2013). Metabolomic analyses of the supragingival biofilm paired with machine learning–based classifiers provide additional biological informed approaches for better understanding and phenotyping early childhood caries and its subtypes (Heimisdottir et al. 2021). Arguably, using deep or granular phenotyping of biologically informed traits in large diverse populations can ultimately provide insights into the molecular basis of dental caries, periodontal disease, and their postulated subtypes (Divaris 2019). Understanding the molecular basis of pathological conditions can provide additional knowledge for the evaluation of risk, disease prognosis, and potential targeting of interventions based on these factors.

## Periodontal Disease

Our understanding of periodontal disease and dental caries is hampered by many of the same issues. Dr. Jim Beck reviewed what is known of the genetic determinants for periodontal disease. Investigations supplementing clinical measures of periodontal health and disease with inflammatory and biofilm measures can help define disease determinants and mechanisms and elucidate associations between severe chronic periodontal disease and other conditions (e.g., cardiovascular; Offenbacher et al. 2018; Beck et al. 2020). Information characterizing the health and disease attributes for each tooth can serve as a basis to classify the periodontal phenotype of an individual (periodontal profile class [PPC]; Beck et al. 2018). To categorize study participants into more homogenous groups for further study, this information can be agnostically interrogated using a data-driven latent class analysis. Using this approach, the PPC-classified populations were seen to be reasonably well-aligned with criteria defined in the recent World Workshop on periodontal disease (Morelli et al. 2017; Caton et al. 2018) as well as being more heritable, indicating that PPC has a stronger biological underpinning than the World Workshop classification (Agler et al. 2019). Understanding biological processes involved in periodontal disease and being able to assess them could allow individuals to be categorized into clinically and biologically similar clusters that help predict disease trajectory and could be useful for providing more directed application of therapeutics and interventions (Divaris et al. 2020).

## Dental and Craniofacial Phenotyping

There are thousands of pathologies that have a genetic etiology, and many known syndromes are associated with changes in dental, oral, and craniofacial structures. These phenotypic changes can be the initial presenting features and easily recognizable or they can be subtle and difficult to discern. Dr. Tim Wright illustrated the diverse clinical phenotypes that arise from mutations associated with genes important for tooth and enamel formation. The range in clinical appearance, coupled with compositional and structural changes, can also vary depending at least partially on the alteration in protein composition and/or structure and the resulting change or loss of function.

Drs. Ophir Klein, Benedikt Hallgrimsson, and Janice Lee addressed the use of craniofacial phenomics and deep phenotyping as critical complements to genome sequencing for diagnostics and health care. New imaging techniques continue to improve our ability to quantitatively analyze morphology and add to our understanding of developmental biology and quantification and assessment of clinical phenotypes (Cole et al. 2017; Hallgrimsson et al. 2020). Facial shape variation is highly polygenic. The human face presents, therefore, diverse and useful phenotypes for evaluating genetic contributions and identifying causes of dysmorphology (Xiong et al. 2019). Three-dimensional imaging coupled with machine learning–based classification for specific heritable conditions provides a high rate of recognition and specificity sufficient to discriminate related but unaffected family members (Hallgrimsson et al. 2020). The identification and validation of 3-dimensional craniofacial landmarks using cone-beam computed tomography scans provides additional phenotyping to assess growth and development, diagnostics, and also guide surgical and nonsurgical craniofacial treatments (Liberton et al. 2019).

## Microbiome and Multi-omics Phenotyping

Humans and their resident microorganisms have interacted over millennia, influencing human traits and genetics and microbial responses (Suzuki and Ley 2020). In session 4, the concept of deep phenotyping microbial communities was explored. Dr. Michael Gilmore discussed *Enterococcus faecalis*, a frequent colonizer of the gastrointestinal tract and persistent pathogen in pulpal infections. He highlighted key events during evolutionary time that drove *E. faecalis* speciation and diversification of function. Through genetic evolution, the basis for adaptation to native habitats could be better understood (Lebreton et al. 2017). Through time, an impervious cell envelope of enterococci evolved to contend with cycles of host gut colonization, excretion as fecal deposits in the environment, and recolonization of new hosts. About 425 million y ago, a time coincident with the emergence of land animals, the enterococci diverged genetically from a common ancestor with vagococci. Able to colonize the gut of land animals, the enterococci typically have genomes of about 3 Mb, which leaves them auxotrophic for typically several amino acids and vitamins. However, the genome sizes vary widely, from about 2.2 Mb for species that are highly adapted to a specific nutritionally accommodative niche to about 5.6 Mb, which enables them to colonize niches, possibly multiple, that have yet to be characterized. In contrast, the vagococci are restricted in their ability to survive desiccation and starvation and do not show the hallmark intrinsic resistance of enterococci. In the antibiotic era over the past 70 y, multidrug-resistant strains of *E. faecalis* and *E. faecium* have emerged under repeated cycles of new antibiotic introduction and selection, which favored the emergence of strains lacking CRISPR protection of the genome, that, as a consequence, are more facile at gaining mobile genetic elements carrying additional antibiotic resistance.

With many of the genes gained by enterococci since diverging from vagococci being novel to the enterococci and of unknown function, Biolog testing (www.biolog.com) has been used to identify 43 specific phenotypes that distinguish enterococci from their ancestors. Identifying phenotypic features as metabolic output facilitates the identification of specific genes, biosynthetic gene clusters, and genetic pathways required to explain, for example, susceptibility or resistance to biocides, β-lactams, desiccation, or starvation (Gilmore 2019). Hence, the characterization of complex phenotypes of E. faecalis and other enterococci is essential to identifying the clusters and networks of genes necessary to create the phenotype. Deep phenotyping drives the elucidation of complex genetic pathways.

In Dr. Edlund’s laboratory, scientists are creating comprehensive phenotypes that describe children prone to dental caries based on a multi-omics approach (Baker et al. 2021). By interrogating saliva samples, the team has identified members of the microbial community and immune factors that co-occur with deep dentin caries. The microbial community disease phenotype includes features such as glucan production, robust biofilm formation, and acid tolerance and production (Aleti et al. 2019). In contrast, healthy salivary samples contain antimicrobial proteins/peptides, vitamins, and pH-neutralizing capacity (i.e., higher levels of arginine). By studying the metabolomic output of the community (metabolomics) in the context of health and caries occurrence, the metabolic potential can be described in terms of primary and secondary metabolites. Primary metabolites include arginine, whereas secondary products might reflect antibiotic production.

The production of antibiotics by bacterial members of the community suggests novel means of interspecies antagonism that might govern community diversity. By sequencing and application of machine learning, biosynthetic gene clusters (BCGs) can be characterized to define an entire pathway for the synthesis of an antibiotic. Indeed, the oral microbiome may contain as many as 5000 BCGs, suggesting an enormous undiscovered reservoir of naturally occurring antibiotics.

By analyzing actively transcribed BCGs in vivo, health and caries can be discriminated. A BCG encoding the biosynthetic pathway *muc* contains genes for a hybrid polyketide synthase and nonribosomal peptide synthetase, which are conserved in many strains of *Streptococcus mutans* isolated worldwide (Tang et al. 2020). Among the products are the antibiotics reutericyclin and mutanocyclin. Using reutericyclin, for example, S. mutans can antagonize neighboring species to alter a healthy plaque community to create a microbiota that is dysbiotic and cariogenic. By characterizing the metabolome and other complex features of oral microbial communities, Dr. Edlund’s group is elucidating the genetic determination of the basis of a disease-causing S. mutans phenotype.

## Conclusions

**Figure 3.**
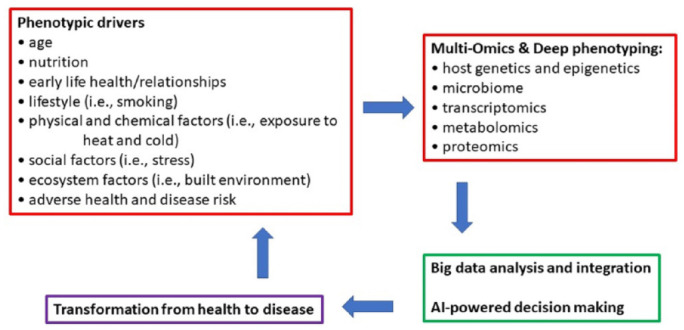
Evaluation using multi-omics, deep phenotyping, and diverse data sets informs a cycle of discovery directed at understanding health and disease and supporting the evolution of health care toward predictive and precision approaches.

Using multi-omics coupled with light and deep phenotyping approaches is expanding our understanding of health and disease determinants. Identifying the diverse drivers and determinants of phenotypes is critical for understanding health and disease and to better inform diagnostics and therapies. The application of tools such as machine learning and artificial intelligence is enhancing our ability to identify important relationships that exist in phenotype determinants and the pathophysiology of disease (Fig. 3). Merging large data sets derived from diverse populations is providing a robust approach for identifying genotype-phenotype relationships and advancing our understanding of the human variation that occurs in specific traits (Shungin et al. 2019; Xiong et al. 2019). Evaluating temporal relationships and heterogeneous progression patterns such as occur with dental caries, periodontal disease, and tumorigenesis provides additional challenges. Temporal phenotyping coupled with machine learning is being applied to help predict clinical outcomes and disease progression (Lee et al. 2020).

## Author Contributions

J.T. Wright, contributed to conception, design, and data acquisition, drafted and critically revised the manuscript; M.C. Herzberg, contributed to conception and data acquisition, drafted and critically revised the manuscript. Both authors gave final approval and agree to be accountable for all aspects of the work.
